# Opportunities for digitally-enabled personalization and decision support for pediatric growth hormone therapy

**DOI:** 10.3389/fendo.2024.1436778

**Published:** 2024-10-15

**Authors:** Paul Dimitri, Paula van Dommelen, Indraneel Banerjee, Riccardo Bellazzi, Marta Ciaccio, Antonio de Arriba Muñoz, Sandro Loche, Azriyanti Anuar Zaini, Ammar Halabi, Merat Bagha, Ekaterina Koledova

**Affiliations:** ^1^ University of Sheffield, Western Bank, Sheffield, United Kingdom; ^2^ Department of Child Health, The Netherlands Organization for Applied Scientific Research TNO, Leiden, Netherlands; ^3^ Royal Manchester Children’s Hospital, Manchester University Hospitals Foundation Trust, Manchester, United Kingdom; ^4^ Department of Electrical, Computer and Biomedical Engineering, University of Pavia, Pavia, Italy; ^5^ Endocrinology Service, National Pediatric Hospital “Prof. Dr. Juan P. Garrahan”, Buenos Aires, Argentina; ^6^ Paediatric Endocrinology, Hospital Universitario Miguel Servet, Zaragoza, Spain; ^7^ Research Area for Innovative Therapies in Endocrinopathies, Bambino Gesù Children’s Hospital, IRCCS, Rome, Italy; ^8^ Department of Paediatrics, University of Malaya, Kuala Lumpur, Malaysia; ^9^ Global Digital Health, Ares Trading SA, Eysins, Switzerland, an affiliate of Merck KGaA, Darmstadt, Germany; ^10^ Tiba Medical Inc., Beaverton, OR, United States; ^11^ Global Medical Affairs Cardiometabolic & Endocrinology, Merck Healthcare KGaA, Darmstadt, Germany

**Keywords:** decision support, digital, growth hormone, pediatric, technology

## Abstract

Smart technologies and connected health are providing opportunities for improved healthcare for chronic conditions. Acceptance by healthcare professionals (HCPs) and patients is crucial for successful implementation. Evidence-based standards, technological infrastructure and regulatory processes are needed to integrate digital tools into clinical practice. Personal health records provide continuity and aid decision-making, while machine-learning algorithms may help in optimizing therapies and improving outcomes. Digital healthcare can negate geographical barriers, enabling patients in remote areas to access specialist endocrine expertise. We review available and developing digital tools to manage care for patients requiring growth hormone (GH) therapy for growth failure conditions. GH is most often administered via daily injections over several years; continuous adherence is necessary but may become insufficient. Future development and integration of electronic platforms for GH therapy requires involvement of all stakeholders in design-thinking approaches and human-factor testing. Growzen Connect is an innovative digital ecosystem designed to increase the management and monitoring of GH therapy, comprising the easypod device and connected mobile apps. It provides a real-time overview of a patient’s therapy, including adherence and growth response, which aids decision-making by HCPs and empowers patients to engage in their therapy journey. Incorporating prediction models for adherence and growth in the ecosystem helps patients build treatment habits and allows issues to be addressed in a timely fashion. A connected ecosystem for GH therapy can enhance outcomes and empower patients, fostering a collaborative and patient-centered approach that is more proactive, beyond the traditional clinic-based approach.

## 1 Introduction

Smart technology and connected health solutions have rapidly brought about significant improvements in the management of long-term health conditions (1–4). In child health settings, numerous opportunities have emerged, ranging from more accurate screening and diagnosis to remote communication and management, enhanced education, and the availability of reliable data (5–9). To fully realize these opportunities, seamless integration is necessary, bridging the gap between hospital and home settings, and utilizing diverse digital solutions to form a digital ecosystem that includes artificial intelligence (AI), virtual reality (VR), sensor and assistive technologies, robotics, and mobile apps.

For digital health solutions to be successfully adopted, acceptance from patients, families, and healthcare professionals (HCPs) is crucial (10). Additionally, industry support is needed to ensure continuous maintenance and integration within clinical pathways. Procurement, commissioning, and reimbursement by health services and systems are also essential to facilitate widespread implementation. However, despite the progress in health technology made to date, there remains a need to establish evidence-based standards, develop technological infrastructure, define regulatory processes, and to seamlessly integrate digital tools into clinical practice (11, 12).

### 1.1 Adopting a holistic approach

A digital health holistic approach refers to the integration of various strategies within a connected ecosystem to provide comprehensive and patient-centered care. It recognizes that health is influenced by multiple factors, including physical, mental, emotional, and social dimensions, and aims to provide digital tools to support the overall well-being of individuals. Digital health solutions enable patients to take ownership of their health, track their progress, and engage in shared decision-making (13). They facilitate seamless collaboration and coordination among specialist clinicians, enabling interdisciplinary care teams to work together, share information efficiently, and ensure a comprehensive understanding of the individual’s health needs (14). Available data sources that facilitate clinical decision-making are fundamental to providing the best outcomes for patients. Clinical decision support (CDS) refers to the use of technology, data, and evidence-based guidelines to provide actionable information to assist HCPs and patients in making timely, informed point-of-care decisions that enhance patient safety, reduce medical errors, and promote evidence-based medicine (15).

## 2 Digitally-enabled holistic care in growth hormone therapy

A series of global meetings of pediatric endocrinologists took place from November 2021 to October 2022 to explore their opinions on the current and future digital landscape for support and management of children and young people with growth disorders, building on the framework developed from previous meetings (16). Bringing together experts from around the world provided perspectives across multiple healthcare systems. Participants explored the current Growzen digital ecosystem (17) and other digital tools to support patients with growth disorders requiring GH therapy. Participants examined the potential of emerging technologies, such as AI and big data analytics, to facilitate tailored and individualized care during the GH journey. Participants recognized the importance of involving patients and their families in the decision-making process, and discussed how digital tools can facilitate better communication, education, and engagement. This paper sets out the views gathered from these meetings and considers the future opportunities to enhance and personalize healthcare for children and young people requiring GH therapy, from diagnosis through to entering into adult healthcare.

### 2.1 The current GH journey

GH produced by the pituitary gland plays a vital role in promoting growth, development, and overall metabolic function. Recombinant human GH is used to treat GH deficiency and childhood growth disorders, and licensed indications are summarized in **Table 1** (18–22). In all pediatric indications, the aim is to achieve the best height outcome within the bounds of a safe dose. The ‘GH journey’ typically begins with the identification of growth-related concerns, which may be through automated monitoring of health records, as seen in Finland (23), or following observations by parents, caregivers, teachers, or HCPs, who have identified slow growth, abnormal body proportions, dysmorphic features, delayed puberty, or other signs of GH deficiency or related conditions (24, 25). A specialist in pediatric endocrinology will assess medical history, physical examination, and specific tests to determine the child’s growth pattern, hormone levels, and overall health, and ruling out underlying causes (26). Once the diagnosis is confirmed, the pediatric endocrinologist will determine if GH therapy is warranted and, if indicated, will discuss the treatment plan with the child’s parents or caregivers, explaining the benefits, potential risks, and expected outcomes.

**Table 1:** Approved indications for growth hormone therapy.

Indication	Characteristics	Aim of GH therapy
**Pediatric GH deficiency** (18)	Growth retardation, delayed physical development, and impaired metabolism	Stimulate linear growth, increase adult height, and improve metabolism
**Turner Syndrome** (19)	Girls with this genetic disorder frequently have short stature	Improve growth rate, and adult height
**Idiopathic Short Stature** (20)	Significantly shorter than peers, with no identifiable cause, and diminished growth potential	Improve growth rate, and adult height
**Chronic Renal Insufficiency** (20)	Children with chronic kidney disease may experience growth failure	Promote growth and improve their overall well-being
**Prader-Willi Syndrome** (20)	Genetic disorder, characterized by multiple physical, cognitive, and behavioral abnormalities	Improve growth, body composition, and muscle strength
**Small for Gestational Age** (21)	Born small for gestational age and remain short by the age of 4 years	Improve growth velocity and catch-up growth
**Adult GH Deficiency** (22)	Impaired body composition, physical function, metabolism, and quality of life	Increase muscle mass and strength, decrease body fat, increase bone density, and improve quality of life

GH therapy is usually administered via daily subcutaneous injections, with the appropriate dosage prescribed according to the child’s specific condition, age, weight, and other individual factors. Parents or caregivers will receive training on how to administer the injections, and ongoing support will be provided by the healthcare team. The response to treatment will be monitored at regular follow-up visits, evaluating growth parameters, hormone levels, and bone age, and monitoring any potential side-effects. GH therapy is a long-term treatment, lasting several years until the child reaches adult height. A pivotal period for instilling habits and ensuring treatment adherence occurs during therapy initiation, when specific concerns of patients and their parents can be addressed (27, 28). Implementing early behavior change strategies can establish a foundation for future adherence, facilitated by improved knowledge and education, through enhanced digital communications. The treatment plan may be adjusted over time to optimize the child’s growth and well-being, and the pediatric endocrinologist will provide guidance on transitioning to adult endocrinology care if necessary. Throughout the GH journey, regular open communication between the child, parents, caregivers, and the healthcare team is crucial to ensure the best possible outcomes for the child.

## 3 Current digital healthcare for children on GH therapy

### 3.1 The GH digital ecosystem

Similar to many other medical specialties, telemedicine, remote monitoring, and virtual care have gained prominence in GH therapy (29). Remote consultations, video conferences, and secure messaging platforms have allowed healthcare providers to connect with patients and assess their progress without the need for in-person visits. This provides convenience, reduces the burden of travel, and increases access to specialized care, particularly for patients in remote or underserved areas. Care is needed to ensure accurate height and weight measurements are taken by parents and carers for remote consultations; however, without novel technologies for accurate height measurements (see section 4.1), visits to the clinic are still required. Currently, the Growzen Connect™ system is the most advanced digital platform to support children and caregivers through the GH journey, designed to increase the management and monitoring of GH therapy for children with growth disorders. The digitally connected ecosystem supports the easypod^®^ fully automated, user-friendly electronic injection device for the subcutaneous administration of GH (30). The Growzen Connect ecosystem consists of three main components: the easypod device, a mobile application (Growzen Buddy™), and a cloud-based platform. The easypod injection device automatically delivers the prescribed dose of GH and is equipped with features to adjust the injection time and depth, and needle speed and type, ensuring accurate and consistent administration (30–32). The device also incorporates a display screen that provides feedback and guidance during the injection process. The mobile application, compatible with smartphones and tablets, serves as the interface between the device and the cloud-based storage platform, enabling patients or caregivers to wirelessly synchronize data, including information on injection timing, dosing and history.

Stored data in the cloud-based platform provides a comprehensive overview of the patient’s GH therapy, including adherence patterns, and growth response. Data relating to adherence is automatically generated when GH is administered, and users can also directly input the height and weight of the patient into the online Growzen Buddy app. Data uploaded to the cloud can be reviewed and analyzed by HCPs and authorized caregivers, to monitor treatment progress. The Growzen Connect system offers several benefits for all stakeholders involved in GH therapy. For patients and caregivers, it simplifies the process of managing injections, tracks therapy adherence, and provides reminders for upcoming doses, thus promoting patient engagement and empowering them to actively participate in their treatment journey. HCPs benefit from the system’s ability to visualize real-time data, which facilitates more personalized and evidence-based decision-making. They can remotely monitor treatment adherence, identify any issues or deviations, and intervene promptly when necessary. The system also streamlines communication between clinicians and patients, enabling timely feedback, guidance, and support. To demonstrate the value of the Growzen ecosystem on patient adherence to GH therapy, the impact of the smartphone app was evaluated in a cohort of Argentinian patients who had at least one year of adherence data and were aged between 2 and 18 years at treatment start. Adherence data were available for 515 patients before implementation of the Growzen Buddy app in the country and for 315 patients after implementation. Before implementation, the proportion of patients with optimal adherence (defined as ≥85% based on GH injected versus GH prescribed) during the first year of treatment was 68%. Following implementation of the app, optimal adherence increased to 82%, with higher adherence seen in those who actively entered height measurements into the platform (33).

### 3.2 Digital educational tools and patient/caregiver support

Patient support programs have been integrated, in accordance with local regulations, in the Growzen Connect ecosystem. The Growzen Buddy app enables users to self-monitor growth, build a routine, and improve adherence. It contains educational resources, videos, and tools to provide guidance and information specific to the GH therapy journey. This helps patients and caregivers understand the rationale for GH therapy, with information on the medication, administration techniques, potential side effects, and strategies for effectively managing the therapy (34). The Growzen Buddy app facilitates communication between patients, caregivers, and HCPs, offering channels for reaching out to clinical support specialists for guidance, addressing concerns, and receiving timely feedback. It can also foster a sense of community among patients and parents/caregivers by providing opportunities for connecting with others going through similar experiences, allowing sharing of knowledge and mutual support (35). Additionally, a patient support program called TuiTek™ incorporates a short satisfaction questionnaire followed-up by nurse-led calls, which use behavior change techniques and implement motivational interviewing principles, tailoring support to the individual needs of each patient and caregiver (36, 37). TuiTek has been shown to be efficient in alleviating anxieties of caregivers and patients across the therapy journey, in different regions around the globe.

HCPs also need information in an easily accessible digital format to further their education, and to ensure consistency across healthcare systems. Massive Open Online Courses (MOOC) are a type of digital educational platform that offer a wide range of subjects to a large number of participants simultaneously, often numbering in the thousands or even tens of thousands, making online education accessible to a diverse and global audience (38). Learners have flexibility to access course materials and complete assignments at their own pace, allowing them to balance learning with other commitments. Many MOOCs incorporate interactive elements such as discussion forums, peer-reviewed assignments, and quizzes to engage learners. Recently, a MOOC has been developed entitled ‘*Tools to Support Growth Disorders Through Telemedicine in the Post-COVID Era’* available on the FutureLearn platform. This course explores how advances in technology and changes in healthcare delivery have influenced the diagnosis and treatment of growth disorders, with a focus on remote healthcare methods (38). Subscribers can access learning on digital literacy in diagnosis management and examine the ways eHealth can support individuals with growth disorders, based on case studies from pediatric patients requiring GH treatment.

## 4 Future perspectives: digital tools to support healthcare professionals

Regarding future digital personalized healthcare, several considerations have been identified as important for the care of patients with growth disorders, summarized in **Table 2**. HCPs need to provide expertise and guidance for effective use of digital tools for personalized treatment and support, and evidence of efficacy will be crucial for commissioning decisions to ensure that the most beneficial tools are incorporated into clinical practice. Integration with EHR and interoperability among these tools are crucial for seamless and holistic care (39, 40) and to enable data analytics to identify trends, patterns, and digital biomarkers related to disease awareness and treatment coherence, education, mental well-being, and quality of life (41).

**Table 2:** Important considerations in development of digital healthcare tools.

**HCP contact will remain crucial**: While digital tools can enhance accessibility and convenience, they should complement rather than replace human interaction; the role of HCPs in patient care cannot be undermined.
**Generating evidence of efficacy is paramount**: As digital tools become more prevalent in healthcare, it is vital to gather robust evidence of their effectiveness and impact through rigorous research and clinical studies.
**An ecosystem of tools is necessary**: To address the multifaceted needs of personalized healthcare, an ecosystem of digital tools is required to cover a wide range of functionalities, such as remote monitoring, personalized education, behavioral interventions, mental health support, and quality of life assessment.
**Data can be utilized to refine digital systems and understand patients**: By analyzing data collected from digital tools, insights can be gained to better understand patients’ needs, preferences, and health outcomes, informing the development of more personalized and effective interventions.

### 4.1 Future height measurement

In the context of pediatric GH therapy, tracking height is crucial to monitor the response (42). Presently, the Growzen Buddy app incorporates monthly prompts to measure and input both height and weight data. The app presents growth information visually through graphs, accessible to patients, caregivers, and HCPs, fostering improved engagement (43). At present, height data must be derived from manual height measurements performed by caregivers at home and the data entered into the app. Whilst the app provides guidance on optimal measurement practice, height measurement for those who are not well-trained has the potential for inaccuracy through user error (44). To address this, a new digital technology is being developed to measure height using augmented reality (AR) within the camera of a mobile device, enabling parents/caregivers to measure their child’s height at home, providing a more accurate, convenient and accessible way to track growth progress. Currently, the precision and accuracy of the height measurement tool are being assessed by comparing AR measurements at home with those obtained in a clinical setting. By implementing this home-based tool, evaluation of growth response could be done more efficiently and frequently, enabling timely adjustments to GH dose, whilst ensuring continued engagement with the therapy process. In the initial period of the patient journey, early diagnosis could be facilitated by the ability to measure height and weight progressively at home or in the community, and analytical models may be implemented to support the earlier recognition of growth disorders. Rapid assessment through online digital systems will facilitate quicker referral to a pediatric endocrinologist, facilitating earlier investigation, diagnosis and intervention.

### 4.2 Predicting response to therapy

The Growzen Connect ecosystem has become a valuable source of data on GH administration and height in pediatric GH therapy. Big data analytics will be used to develop prediction models on future therapy adherence, thus identifying those most in need of support. These prediction models utilize a traffic-lights approach to report to the HCP, with green reflecting good adherence, and red indicting poor adherence and the need for intervention (41, 45). Given their simplicity, traffic-light systems have become widely recognized by both HCPs and patients, fostering better understanding and facilitating joint decision-making by providing a platform for discussions about potential issues.

Two prediction models have been developed to monitor GH adherence and could be incorporated into a future digital ecosystem to provide CDS: the first predicts adherence in the next 9 months based on an individual’s adherence data from the initial 3 months (46); the second is more flexible, using the prior 6-monthly adherence data to predict adherence in the next 3 months and allows for early identification of adherence issues as the child grows older and starts self-medication (47). The predictions from the first model have shown accuracy of up to 77% for sub-optimal adherence and ≥80% for optimal adherence (46, 47). Factors identified as influencing future adherence include age, sex, data transmission frequency, device settings, and changes in dose (46).

Predicting future growth is also fundamental for patients on GH therapy, aiding the HCP in managing therapy and expectations, but also providing an incentive towards better adherence if patients and caregivers are shown the outcome from optimum adherence. The most recently developed method to predict future response to therapy uses a curve-matching technique that predicts growth using data of patients whose growth curves resemble those of a new patient (48). The Growzen ecosystem can collect information such as the diagnosis and the adherence to GH therapy. Results from curve matching in combination with adherence may encourage therapy persistence. It also enables HCPs to identify any deviations from the predicted response, which can help verify the accuracy of the diagnosis and appropriateness of the GH dose (41). The introduction of the new easypod device, which captures and transfers adherence data in real-time, will facilitate rapid, accurate and whole-scale use of prediction methods to improve GH therapy and patient support (31).

### 4.3 Digital patient reported outcome measures

Incorporating patient reported outcome measures (PROMs) within a digital ecosystem can provide a more comprehensive and holistic understanding of the impact of medical interventions. PROMs capture the physical, emotional, social, and psychological effects of a medical condition or treatment from the patient’s viewpoint (49–52). By valuing and incorporating the experiences of patients, healthcare systems can improve care delivery, enhance patient satisfaction, and promote better health outcomes, through shared decision-making and discussions. The development of a digital ecosystem to gather information about the wider issues of patients on GH therapy could empower HCPs to deliver care that aligns with patients’ goals, preferences, and values. For example, patients on the GH therapy journey may encounter mental health issues, anxiety or depression, and additional digital tools could support them whilst they access relevant mental health support from trained professionals. In this context, AI-driven, reliable and clinically-approved virtual assistants, accessed through signposting and healthcare support, could be integrated into a GH administration connected ecosystem to provide rapid online information relating to the condition, wellness tips, healthy lifestyle suggestions, nutrition, exercise, and mental well-being. By assessing outcomes that matter most to patients, such as pain reduction, improved quality of life, and functional ability, HCPs are able to gauge the actual effectiveness of treatments beyond standardized clinical metrics (49). Moreover, patient-reported data fuels research initiatives and informs future digital solutions that directly address patients’ needs, providing insight into healthcare disparities among different demographic groups, and information for policymakers when designing healthcare policies and allocating resources.

## 5 Future perspectives: digital tools to support patients and caregivers

During the global advisory boards, the pediatric endocrinologists considered the existing digital tools utilized in GH therapy and potential future enhancements required to provide better support to patients and caregivers. All recognized that simplicity holds immense importance for fostering engagement among both patients and clinicians. The principle of simplicity of digital tools revolves around creating user-friendly interfaces and intuitive designs, ultimately leading to seamless usage, widespread adoption and meaningful interactions. Furthermore, building motivation for long-term care is crucial to foster continued engagement with GH therapy to provide favorable physical and mental health.

### 5.1 Adopting a personalized and patient-centric approach

As AI technologies advance, machines can learn about individual patient needs, enabling more tailored, patient-centric care (46, 53). Personalization may be influenced by various factors, including demographics, stage of the GH therapy journey, and individual patient characteristics. Considering the age and developmental stage of the patient is crucial; some stories, education and guidance may be more relevant and relatable to younger children and their parents, while others may be better suited for teenagers who are self-administering their treatments. In the future, AI may assist in personalizing treatment plans by considering GH dose adjustment based upon the individual characteristics and lifestyle of the patient (12). Predictive analytics algorithms may also forecast potential health issues, treatment response, and treatment complications based upon symptoms, by identifying patterns and correlations within the patient’s data, enabling earlier intervention and prevention strategies (32). Data acquisition may need to extend to wearable devices that provide additional patient physiological health data to the GH digital ecosystem (54, 55).

Similar to other clinical areas, AI in the care of growth disorders poses challenges related to data privacy, algorithm transparency, and the need for interdisciplinary collaboration between HCPs, data scientists, and ethicists to ensure responsible and effective implementation (56–59). Patients and caregivers should be fully informed about how their data will be used in AI applications and should have the right to give informed consent for its use. Transparent communication is necessary to ensure patient understanding and compliance; thus, establishing clear regulatory frameworks that promote innovation while safeguarding patient safety and privacy are essential, and AI-driven interventions must be monitored for long-term efficacy and safety.

### 5.2 Enhancing clinical decision support

Monitoring of treatment progress by tracking pertinent parameters such as growth charts, hormone levels, or other markers of therapeutic response, empowers HCPs to make data-driven decisions to fine-tune treatment as necessary. In the future, the digital system could provide guidance on recognizing and addressing potential side effects or complications linked to GH therapy. This includes offering advice on when patients should seek medical attention and steps to undertake if adverse events arise. Furthermore, through regular monitoring, the system could offer advice on nutrition, physical activity, and other lifestyle facets that contribute to optimal growth and overall health during GH therapy. This, in-turn, empowers patients and caregivers to make well-informed lifestyle choices that align with their treatment objectives.

### 5.3 Building habits for therapy engagement

Digital nudging techniques can effectively support and promote patient and family engagement by utilizing strategies that gently push individuals towards desired behaviors (60–63). These can take the form of notifications, messages, or visual cues, presented through mobile apps or online platforms. Digital nudges should be tailored to the individual’s specific needs, preferences, and circumstances. By collecting data and information about the user, such as their health goals, habits, or demographic factors, personalized nudges can be delivered, increasing their relevance and effectiveness (63, 64). In the GH digital ecosystem, nudges should be delivered at strategic moments to maximize their impact, for example, at the appropriate time for preparation and administration of each dose. Prompts to engage in physical activity could also be provided during periods when the individual is most likely to be sedentary, to improve response to GH therapy, and overall health and well-being. Digital nudges should utilize positive reinforcement techniques, such as rewards, recognition, or virtual badges, as incentives for adopting and sustaining healthy behavior. These reinforcements can help to engage children and young people and encourage them to continue making positive choices.

### 5.4 Digital peer-to-peer support in GH therapy

Digital peer-to-peer support refers to platforms and technologies to facilitate interactions and connections among individuals facing similar health challenges. This harnesses the power of online communities to provide mutual encouragement, information sharing, emotional support, and experiential insights, in turn improving therapy adherence and clinical outcomes (65). Digital peer groups provide a space for patients and caregivers to express their feelings, fears, and concerns openly, creating a sense of belonging and reducing feelings of isolation. Engagement with peers empowers individuals to take an active role in their healthcare decisions, improving resilience and helping inform choices about treatment options and strategies. Participation in digital peer support groups could also lead to improved emotional well-being, increased self-efficacy, and enhanced overall health outcomes, indicating relevance in a GH therapy ecosystem.

Despite the value of peer-to-peer support in GH therapy, there are potential practical challenges in implementation. Digital peer support groups are typically not monitored or moderated by medical professionals, and the information shared in such groups may not always be accurate or evidence-based. Participants might offer advice based on personal experiences that may not be applicable to everyone, potentially leading to misinformation or conflicting and possibly harmful advice. While peer support can provide emotional relief, it cannot substitute professional mental healthcare; mental health apps currently available mostly offer only basic features and suffer from privacy challenges (66). Interactions in online groups might exacerbate emotional distress or contribute to negative emotions, and sharing personal health information online may compromise privacy and security because participants may unintentionally reveal sensitive details that could be exploited by malicious actors.

Some participants may join online groups with the intent to promote specific products, services, or agendas, which can affect the authenticity of interactions and advice. Participants might engage only with those who share similar perspectives, limiting exposure to diverse viewpoints and potentially reinforcing biased beliefs. Individuals with limited health literacy might struggle to interpret and navigate the information shared in online groups, leading to confusion or misunderstanding, and some individuals may disengage after a short period, leading to incomplete discussions and a lack of continuity. To mitigate these issues in the future, digital peer-to-peer support platforms should consider implementing proper moderation, providing evidence-based information, encouraging respectful and empathetic communication, and offering resources to connect participants with professional medical guidance when needed, thus enhancing the value of interactions that may improve care of patients on therapy.

## 6 Conclusions: what could the future of GH therapy look like?

The development and integration of digital technologies, information systems, and electronic platforms for the care of patients on GH therapy for growth disorders could lead to a paradigm shift that will improve outcomes, develop optimal treatment strategies, and improve the physical and mental well-being of patients. This can streamline medical processes, drive research and future innovation, facilitate patient engagement, and personalize healthcare. To achieve this, the involvement of all stakeholders is necessary, employing design-thinking approaches and conducting human-factor testing to refine and optimize usability and effectiveness, and to ensure that digital tools are appropriate for the patient’s developmental stage during the GH journey. Digital healthcare potentially breaks down geographical barriers, enabling patients in remote or underserved areas to access specialist pediatric endocrine expertise and services without the need for travel. Capturing and analyzing vast amounts of patient data can enhance data-driven decision-making, enabling HCPs to make informed, evidence-based decisions regarding diagnosis, treatment plans, and preventive measures, without the need for regular face-to-face appointments. Given the ability to monitor and communicate with patients and caregivers more regularly, modifications to care can be more proactive beyond the traditional clinic-based approach. Ultimately, there will be a future paradigm shift from didactic healthcare focused on disease therapy towards shared managed care and responsibility and a more holistic approach to health and well-being.

Generating empirical evidence is crucial for motivating HCPs and patients to adopt and interact with new digital tools, and case studies that report the experiences of patients, caregivers, and HCPs can serve as a means of validation and effectively showcase the advantages of a digitally connected GH therapy ecosystem. The influence on patient outcomes within clinical trials and when establishing real-world evidence for a novel digitally-connected ecosystem will play a central role in affirming the credibility of these tools. This process will foster trust and confidence in emerging systems, thereby encouraging their widespread acceptance across global healthcare environments. While some individuals may gravitate toward comprehensive digital support, others may opt to withhold immediate adoption of digital solutions until there is clear validation, evidence of impact, and a sense of trust. Addressing challenges related to digital illiteracy, digital exclusion, and financial constraints will also prove pivotal in ensuring equitable access to a connected GH therapy ecosystem within the framework of a national healthcare setting. A key step toward resolving these concerns involves the provision of enhanced digital accessibility packages in the future. This strategic approach will be instrumental in tackling these issues effectively.

Overall, digital healthcare is transforming the landscape of GH therapy, through mobile applications, connected devices, big data analytics with AI algorithms, virtual care platforms and generative AI. Patients and HCPs can benefit from personalized, efficient, and convenient care, with improved clinical decision support. These advances not only enhance treatment outcomes, but also empower patients to actively engage in their therapy, fostering a collaborative and patient-centered approach to healthcare.

## Data Availability

The raw data supporting the conclusions of this article will be made available by the authors, without undue reservation.
